# Microencapsulation of Thymol in Poly(lactide-co-glycolide) (PLGA): Physical and Antibacterial Properties

**DOI:** 10.3390/ma12071133

**Published:** 2019-04-07

**Authors:** Zhu Zhu, Tiantian Min, Xueji Zhang, Yongqiang Wen

**Affiliations:** School of Chemistry and Bioengineering, University of Science & Technology Beijing, Beijing 100083, China; zhuzhu@ustb.edu.cn (Z.Z.); 18865513727@163.com (T.M.); zhangxueji@ustb.edu.cn (X.Z.)

**Keywords:** thymol, PLGA, microencapsulation, natural antimicrobial

## Abstract

Thymol has been shown to be a safe and effective broad-spectrum antimicrobial agent that can be used as a food preservative. However, its volatile characteristics and strong odor limit its use in food products. The microencapsulation of this essential oil in biopolymers could overcome these disadvantages. In this work, thymol-loaded poly(lactide-co-glycolide) (PLGA) microparticles were successfully prepared and the optimal encapsulation efficiency was obtained at 20% (w/w) thymol. Microparticles containing thymol presented a spherical shape and smooth surface. Microencapsulation significantly improved the thermal and storage stability of thymol. In vitro release profiles demonstrated an initial fast release followed by a slow and sustained release. Thymol-loaded microparticles had strong antibacterial activity against *Escherichia coli* and *Staphylococcus aureus*, and the effectiveness of their antibacterial properties was confirmed in a milk test. Therefore, the thymol-loaded microparticles show great potential for use as an antimicrobial and as preservation additives in food.

## 1. Introduction

Microbial spoilage in the food processing industry poses risks to consumers’ health, and causes severe economic losses. *Escherichia coli* (*E. coli*), particularly *E. coli* O157:H7, and *Staphylococcus aureus* (*S. aureus*) are contagious bacteria that have been found in ready-to-eat foods including salads, dairy products, and processed meat [1]. To control the growth of contaminant microorganisms and to meet the requirements of consumers for safe, healthy, and convenient food products, a variety of naturally occurring compounds have been used as antimicrobial preservatives, including antimicrobial peptides, plant-derived substances, and enzymes [2].

Thymol is the natural antimicrobial compound in essential oils that are extracted from plants belonging to the *Lamiaceae* family. This compound has been registered in the European Union (EU) flavoring list, and classified as generally recognized as safe (GRAS) by the Food and Drug Administration (FDA) [3]. Thymol has the ability to damage bacterial lipid membranes and has been shown to be an effective broad-spectrum antimicrobial [3,4]. However, there are several limitations to thymol’s application in food products. Specifically, its volatile nature hinders its handling, and its strong odor and flavor can affect the organoleptic acceptability of the treated products. Furthermore, because of its hydrophobicity, the uneven dispersion of thymol in food matrices can limit its contact with microorganisms in aqueous environments, thereby reducing its antimicrobial effects [5]. A practical approach to masking sensory attributes and improving the stability and aqueous solubility of essential oils is to encapsulate the essential oil in biopolymers through microspherification or microencapsulation [6,7,8]. In such cases, the microparticles could protect the essential oil from the outer environment, and control the release of essential oil [9]. 

Poly(lactide-co-glycolide) (PLGA), an FDA-approved polymer, has been widely used in biomedical fields such as drug delivery because of its biodegradability, biocompatibility, and controlled delivery properties [10]. Poly(lactide-co-glycolide) can also be used to encapsulate hydrophobic active compounds, and has shown great potential for improving the effectiveness of delivery in food systems [11,12]. In addition, essential oils including cinnamon bark extract and eugenol were incorporated in PLGA, and exhibited efficient antimicrobial activity [13,14]. However, the microencapsulation of thymol in PLGA has yet to be explored. 

In this study, we microencapsulated thymol in PLGA via an emulsion solvent evaporation method. The morphology, encapsulation efficiency, and loading capacity of microparticles were then investigated. In addition, the compatibility and thermostability were determined using Fourier transform-infrared (FTIR) spectroscopy as well as thermogravimetric (TG) and derivative thermogravimetric (DTG) analysis. Finally, the in vitro thymol release profile in air and solution, antimicrobial activity against foodborne pathogens (*E. coli* and *S. aureus*), and milk preservation potential were assessed.

## 2. Materials and Methods

### 2.1. Chemicals

Poly(lactide-co-glycolide) (lactide/glycolide = 50/50, M_w_ = 60 kDa) was purchased from Jinan Daigang Degradable Biomaterial Co., Ltd. (Jinan, China). Poly(vinyl alcohol) (PVA, M_w_ = 27 kDa) was bought from Macklin Reagent Co., Ltd. (Shanghai, China). Thymol (>98% purity) was acquired from Alfa Aesar Chemicals Co., Ltd. (Beijing, China), dichloromethane (DCM) was purchased from Sinopharm Chemical Reagent Co., Ltd. (Shanghai, China). Phosphate buffer solution (PBS, 0.0067 M, pH 7.2–7.4) was acquired from HyClone^TM^ (Waltham, MA, USA). FilmplateTM *E. coli* and coliform count plates and FilmplateTM *S. aureus* were obtained from Oasis Bio-chemical (Guangzhou, China). Pasteurized milk was purchased from a local supermarket. All of the chemicals were of analytical reagent grade.

### 2.2. Microencapsulation of Thymol

Microparticles were prepared by the single emulsion solvent evaporation method. Briefly, PVA was dissolved in distilled water at 0.5 wt% (water phase, W). The oil phase (O) was then obtained by dissolving PLGA (200 mg) and thymol (20, 30, 40, and 50 mg) in 4 mL DCM, after which the oil phase was added dropwise into the water phase and magnetically stirred for about 6 h to evaporate the solvent. The solution was subsequently centrifuged at 5000 rpm and washed three times with ultrapure water to remove excess PVA and non-incorporated thymol. The microparticles were placed in freezing vacuum drying oven (LGJ-10, Beijing Songyuan HuaxingTechnology Develop Co., Ltd., Beijing, China) at −50 °C for 24 h to evaporate the DCM completely, and were subsequently lyophilized in a vacuum desiccator (DZF-6020, Shanghai Heheng Instrument Equipment Co., Ltd., Shanghai, China) and stored at −20 °C for further analysis. In addition, PLGA microcapsules without added thymol were also prepared and used as a control. The experiment was repeated twice.

### 2.3. Encapsulation Efficiency (EE) and Loading Capacity (LC)

Lyophilized microparticles (5 mg) were completely dissolved in 10 mL DCM solutions under stirring. The amount of thymol was then measured spectrophotometrically (UV-6100S, Mapada, Shanghai, China) at 281 nm using a calibration curve. The calibration curve of thymol was linear in the range of 0–40 μg/mL with a high correlation coefficient (R^2^ = 0.99913). The encapsulation efficiency (EE) and loading capacity (LC) were calculated using the following formula:

EE = amount of thymol entrapped/initial amount of thymol × 100%

LC = amount of thymol entrapped/weight of microcapsules × 100%

### 2.4. Microparticle Size and Morphology

The size and morphology of the thymol-loaded microparticles were characterized on a scanning electron microscope (SEM) (SUPRA55, ZEISS, Oberkochen, Germany). The samples were coated with gold and examined at 1.0 kV. The mean size of the microparticles was determined from three images of the same batch (180 microcapsules each) using ImageJ V1.8.0 software. The size distribution was calculated using Origin Pro2018 software.

### 2.5. Fourier Transform-Infrared Analysis

The FTIR spectra of free thymol, PLGA, and thymol-loaded PLGA microparticles were recorded using an FTIR spectrometer (Nicolet Nexus 670, Waltham, MA, USA) in the range of 500–4000 cm^−1^. Samples were mixed with potassium bromide (KBr) and compressed into a tablet.

### 2.6. Thermogravimetric (TG) and Derivative Thermogravimetric (DTG) Analysis

The thermal properties of thymol-loaded microparticles were investigated using a thermogravimetric apparatus (TA, Netzsch, Selb, Germany) by heating from 25 to 500 °C at a rate of 20 °C/min under a nitrogen atmosphere.

### 2.7. Release Study

To evaluate the storage stability, thymol-loaded microparticles were kept in a humidity chamber for 20 days under two humidity conditions (50% and 90%) at 25 °C; and at a relative humidity of 50% at 4 °C. At regular intervals, 5 mg of microparticles were taken and dissolved into DCM. The amount of thymol remaining in the microparticles was then determined spectrophotometrically as previously described.

The release profiles of thymol-loaded PLGA microparticles in solution were analyzed spectrophotometrically as described by Zhang et al. [15]. Briefly, 40 mg of microparticles were dissolved into 40 mL phosphate buffer solution (PBS, 0.15 M, pH 7.4) with 1% Tween-80. For free thymol release behavior, 10 mg thymol was suspended in the PBS solution. The solution was then separately shaken at 100 rpm. At regular intervals, 5 mL of supernatant was withdrawn and filtered with a 0.22-μm nylon membrane syringe filter for UV absorbance analysis. Thereafter, 5 mL of fresh PBS were added into the tubes and placed back in the shaker. The samples were analyzed at 272 nm, and the thymol content was calculated according to a standard curve. 

### 2.8. Antibacterial Activity

The antibacterial activity of microparticles was evaluated against the Gram-negative bacteria *E. coli* and the Gram-positive bacteria *S. aureus* using the colony counting method. *E. coli* (ATCC 25922) and *S. aureus* (ATCC 25923) were kept at −80 °C in Luria-Bertani broth (LB) with 20% glycerol. Routinely, fresh bacterial cultures were obtained from frozen stocks and were grown in LB medium at 37 °C. The bacterial suspension was adjusted to 10^8^ colony forming units (CFU)/mL by measuring the optical density (OD) spectrophotometrically (OD_600_ ≈ 0.4), then was diluted to approximately 10^6^ CFU/mL. Next, the powder of control microparticles and different amounts of thymol-loaded microparticles was dispersed in the diluted bacterial suspension (5, 10, and 15 mg/mL) respectively and shaken for 24 h. The culture (0.1 mL) was subsequently spread on the LB agar plates and incubated overnight at 37 °C. The colonies were counted using an Automatic Colony Counter (icount 20, Shineso Science & Technology Co., Ltd., Hangzhou, China).

The antibacterial tests were also carried out after adding microparticles to milk. In order to obtain relatively high levels of bacteria, the pasteurized milk was stored for 2 days at room temperature and was naturally contaminated. Briefly, the powder of control microparticles and different amounts of thymol-loaded microparticles was dispersed in 25 mL of naturally contaminated milk (5, 10, and 15 mg/mL) and shaken for 24 h. Then the milk was diluted (1:10) with PBS, after which 1 mL of the diluted solution was added to a tube containing 9 mL of PBS [16]. One milliliter of each sample was placed onto a FilmplateTM *E. coli* and coliform count plate and a FilmplateTM *S. aureus*, according to the manufacturer’s instructions, and then incubated overnight at 37 °C. Plates were then visually assessed for the presence of characteristic colonies (*E. coli* colonies are blue in color, *S. aureus* colonies are purple) using plates that contained 20 to 200 colonies per plate [17]. The experiment was carried out under sterile conditions to avoid artificial contamination.

### 2.9. Statistical Analyses

All statistical analyses were performed with SPSS version 15.0 (SPSS Inc., Chicago, IL, USA). Data from the assay of microparticle size were compared in a Student’s *t*-test. Others were analyzed by one-way ANOVA, and mean separations were determined using Duncan’s new multiple range test. Differences at *p* < 0.05 were considered significant. Each analysis was repeated three times.

## 3. Results and Discussion

### 3.1. Encapsulation Efficiency (EE) and Loading Capacity (LC)

**Figure 1:**
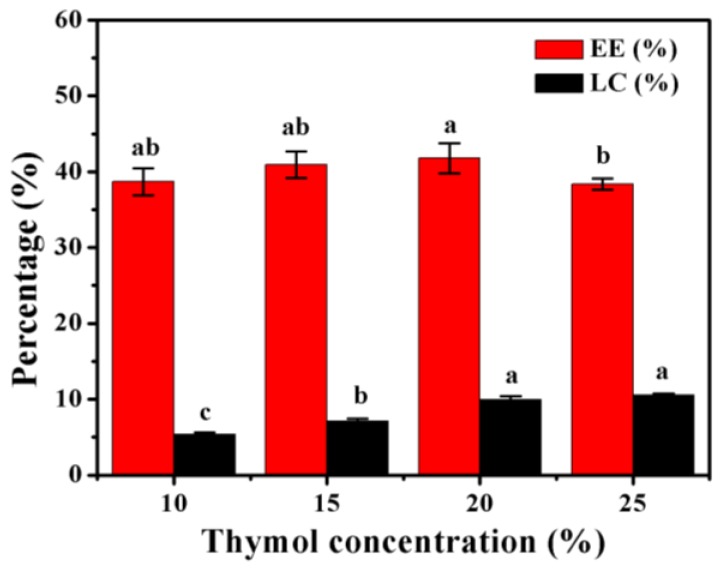
Encapsulation efficiency (EE) and loading capacity (LC) of poly(lactide-co-glycolide) (PLGA) microparticles containing different amounts of thymol. Error bars indicate the standard deviation of the mean. Columns with different letters are significantly different according to Duncan’s multiple range test at *p* < 0.05.

The EE and LC are shown in Figure 1. The EE tended to increase as the thymol content increased to a point and then declined, with the maximum value of 47.19% ± 1.99% being obtained at a concentration of 20% (w/w relative to PLGA). These results were consistent with those of previous studies that showed that an increased amount of essential oil decreases the EE of microparticles [7,8]. The decreased EE could have been associated with the limited capacity of the microparticles.

The microparticles showed an increase in LC as the thymol content increased (Figure 1). These findings are in agreement with those of Benavides et al. [7], who found a direct proportional relationship between LC and the essential oil content. However, LC increased slightly from 9.96% ± 0.47% to 10.54% ± 0.21% when the thymol concentration ranged from 20% to 25%. To achieve optimal efficiency and prevent the unnecessary use of thymol, microparticles containing 20% thymol were selected for further investigation. 

### 3.2. Microparticle Size and Morphology

**Figure 2:**
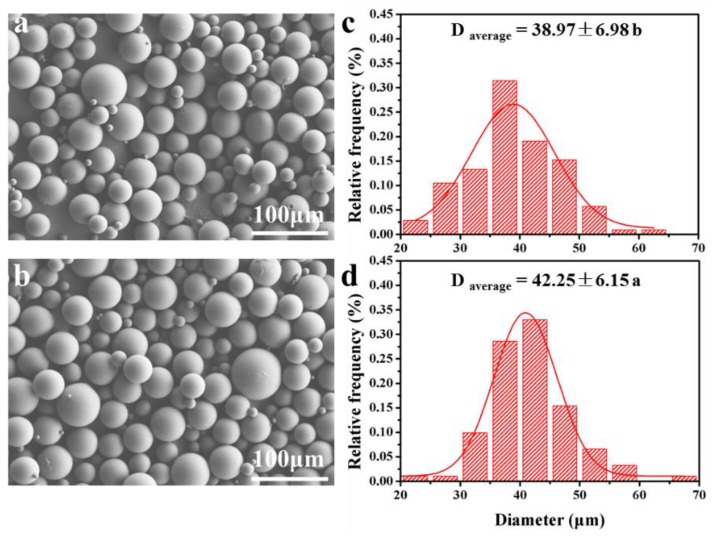
The SEM morphology and size distribution of PLGA microparticles (**a**,**c**) and thymol-loaded PLGA microparticles (**b**,**d**). Different letters indicates significant difference at *p* < 0.05 according to Student’s *t*-test. Data are accompanied by standard deviations of the means.

Figure 2 shows the SEM image and size distribution of unloaded and thymol-loaded PLGA microparticles. All of the microparticles presented a spherical shape and smooth surface with a size distribution of 20–70 μm. Similar results were obtained by Zhang et al. [18], who found that paclitaxel-loaded PLGA microspheres prepared using the single-emulsion solvent evaporation method had a round shape and smooth surface. In terms of size, thymol-loaded PLGA microparticles were slightly larger in diameter than unloaded microparticles. The presence of thymol yielded a more viscous organic phase, making it difficult to disperse the phases in the process of emulsification and therefore led to larger particles. An increase in the size of the microspheres containing essential oil has been observed in various studies [7,13,19]. 

### 3.3. Fourier Transform-Infrared (FTIR) Analysis

**Figure 3:**
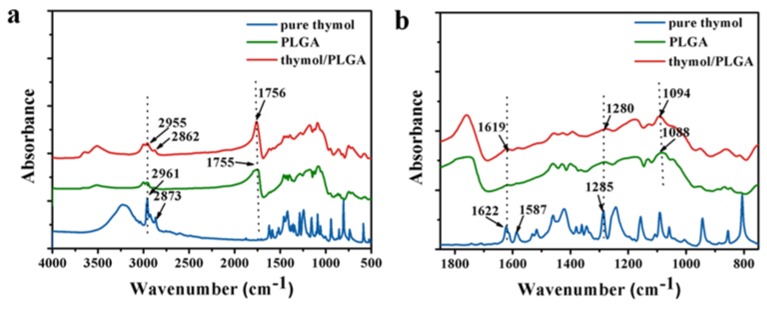
FTIR spectra of free thymol, PLGA, and thymol-loaded PLGA microparticles in the range of 4000–500 cm^−1^ (**a**) and 1800–800 cm^−1^ (**b**).

The FTIR spectra of free thymol, PLGA, and thymol-loaded PLGA microparticles were analyzed (Figure 3). Thymol, which is a natural monoterpene phenol derivative of cymene, is rich in C=C, –CH_3_, and C–O groups. The FTIR spectra of free thymol displayed characteristic peaks at 2873, 1622, 1587, and 1285 cm^−1^, corresponding to anti-symmetric deformation of –CH_3_, stretching vibration of C=C, bending deformation of –CH_3_, and stretching vibration of C–O [20]. Major peaks ascribed to PLGA were observed at 1755 (C=O bending deformation) and 1088 cm^−1^ (C–O–C stretching) [15]. The FTIR spectra of thymol-loaded PLGA microparticles displayed both thymol and PLGA peaks at 2862, 1756, 1619, 1280, and 1094 cm^−1^ with minor shifts in wavenumber. These results confirmed the presence of thymol in PLGA microparticles without chemical changes and were in agreement with the results of previous studies evaluating the microencapsulation of paclitaxel in PLGA [15]. 

### 3.4. Thermal Stability Analysis 

**Figure 4:**
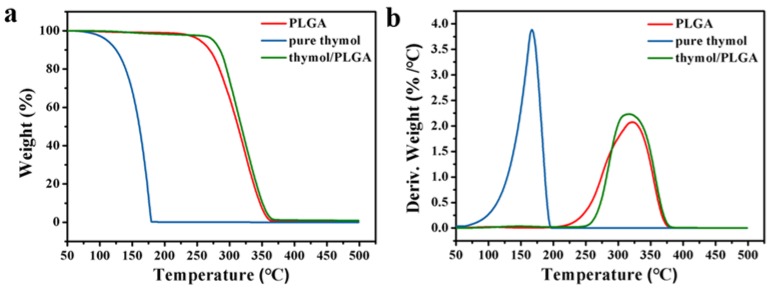
Thermogravimetric (**a**) and derivative thermogravimetric (**b**) of free thymol, PLGA, and thymol-loaded PLGA microparticles.

The TG and DTG curves of thymol, PLGA, and thymol-loaded PLGA microparticles are shown in Figure 4. Thymol evaporation took place between 50 and 175 °C, indicating its volatile nature (Figure 4a). Poly(lactide-co-glycolide) exhibited weight loss at above 225 °C as a result of thermal degradation. The TG curves of thymol and PLGA were similar to those obtained in previous studies [21,22]. Thymol-loaded PLGA microparticles lost weight between 150 and 375 °C, which corresponded to the thermal evaporation of thymol and thermal degradation of PLGA. These results suggested that microencapsulation effectively inhibited the volatility of thymol. As shown in Figure 4b, the weightlessness rate peak of thymol loaded PLGA microparticles was hysteretic relative to that of thymol, which further confirmed that the thermal stability of thymol was significantly improved by microencapsulation. 

### 3.5. In Vitro Release into Air

**Figure 5:**
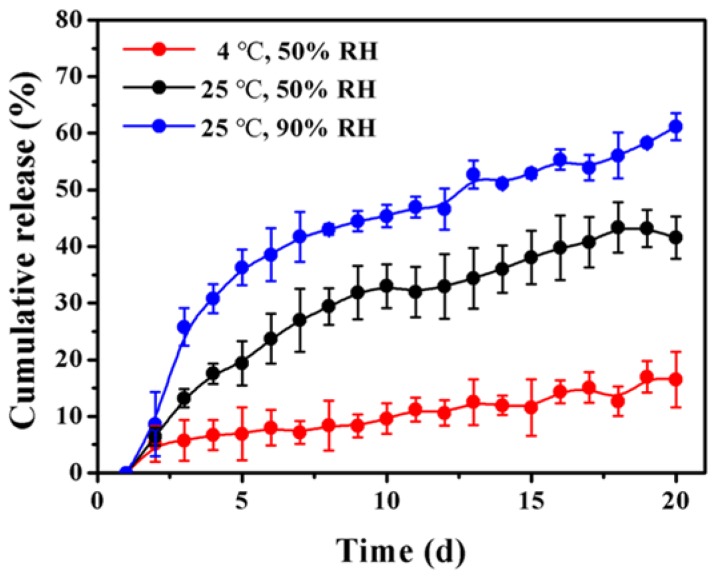
In vitro release of thymol from PLGA microparticles at 25 °C (50% RH), 25 °C (90% RH), and 4 °C (50% RH). Error bars indicate the standard deviation of the mean.

Considering the volatility and structural stability of the thymol (Figure 3), we concluded that the compound released into air at low or room temperature was thymol. The amounts of thymol released from PLGA microparticles at 25 °C (50% and 90% RH, respectively) and 4 °C (50% RH) are shown in Figure 5. The release of thymol from PLGA microparticles increased gradually under all of the conditions with storage time. However, the amount of thymol release at 25 °C was greater than that at 4 °C, which is possibly due to the enhanced polymer mobility [23]. In addition, RH had a significant impact on the release rate of thymol. Specifically, the amount of thymol release was higher at 90% RH than that at 50% RH. A previous study indicated that high RH could decrease the molecular weight of PLGA because of greater hydrogen-bonding with water than the initial hydrophobic polymer [24]. Therefore, the impact of RH on the amount of thymol release could be associated with the enhanced polymer degradation and increased hydrophilicity of thymol. After 20 days of storage at room temperature, PLGA microparticles at 90% RH and microparticles at 50% RH released 61.13 ± 2.38% and 41.54 ± 3.73% of thymol, respectively. In comparison, only 16.50 ± 4.88% of thymol was released from PLGA microparticles at a low temperature at 50% RH. Thymol release in the three conditions was significantly different. These results demonstrated that microencapsulation significantly enhanced the stability of thymol in air. Moreover, the thymol-loaded microparticles could be relatively stable during storage and handling when kept in cool and dry places.

### 3.6. In Vitro Release in Solution

**Figure 6:**
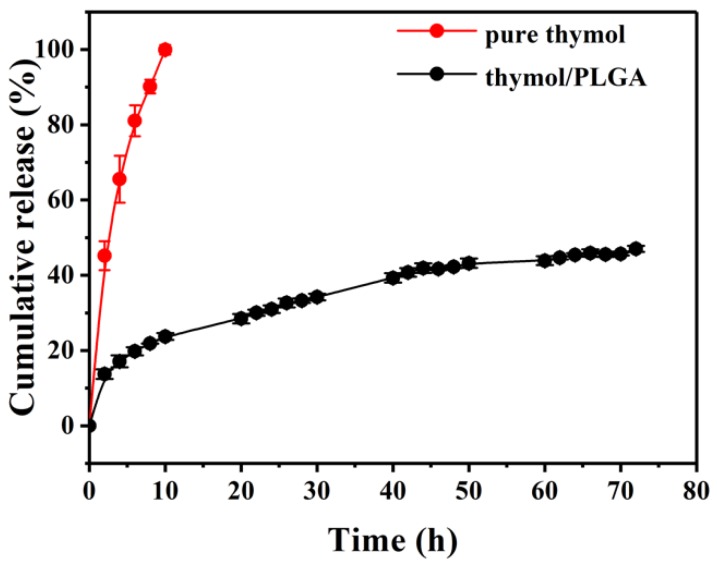
In vitro release of free thymol and thymol-loaded PLGA microparticles in phosphate buffer solution at 25 °C. Error bars indicate the standard deviation of the mean.

The thymol release profiles were evaluated in vitro, and the result over 72 hours of release time is presented in Figure 6. The free thymol release displayed a near-zero-order release behavior, with complete release occurring within 10 hours. The release cure was not a straight line, probably due to the volatility of the thymol. However, the release profile for thymol-loaded PLGA microparticles showed a rapid burst in the first few hours followed by a slow and sustained release with time. Generally, the mechanisms by which active compounds are released from a delivery system can be associated with diffusion of the active compound through the polymer, polymer swelling, and degradation [14]. The reason for the rapid burst effect might be that a portion of the thymol was close to the surface of the particle [25]. The release rate decreases eventually as the substance has to pass through the polymeric matrix to the external environment, which requires more time [13]. After 72 h of incubation, approximately 50% of the thymol was released into the medium. These results were similar to those observed for cinnamon bark extract nanoencapsulated in PLGA obtained by Hill et al. [13]. Overall, PLGA microparticles improved the thymol stability, initially achieved a fast thymol release, and showed a slow and sustained thymol release thereafter.

### 3.7. Antibacterial Activity

**Figure 7:**
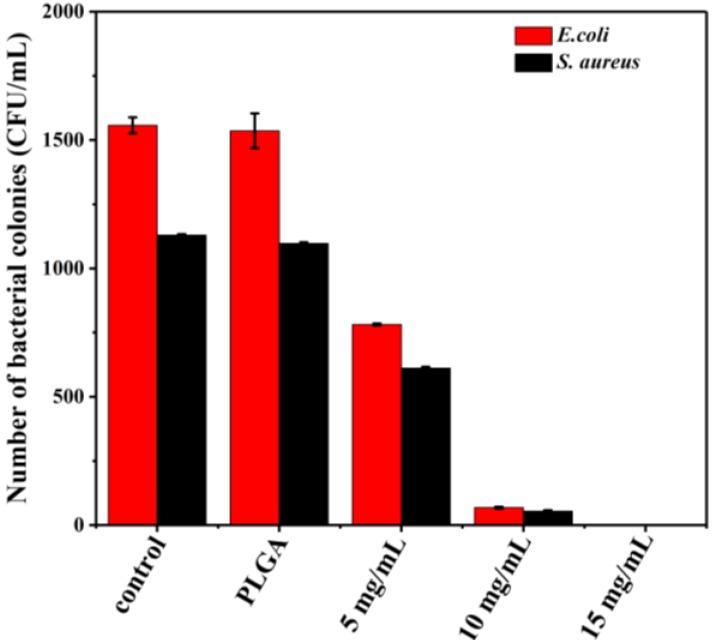
The number of bacterial colonies of *E. coli* and *S. aureus* treated with different amounts of thymol-loaded PLGA microparticles (0, 5, 10, and 15 mg/mL). Error bars indicate the standard deviation of the mean.

Thymol has been shown to possess antimicrobial activity toward a large range of microorganisms [3]. The antibacterial activities of thymol-loaded PLGA microparticles were tested against Gram-negative *E. coli* bacteria and Gram-positive *S. aureus* bacteria via the colony counting method (Figure 7). The antibacterial activity of thymol-loaded microparticles for both bacteria increased as the amount of microparticles increased. The microparticles at a concentration of 15 mg/mL completely inhibited the growth of both bacteria. The inhibition of bacterial growth involves disruption of the cytoplasmic membrane. The phenolic hydroxyl group of thymol increased its hydrophilic ability, which could help thymol dissolve in microbial membranes and damage them [26]. However, there was no difference in bacterial growth between the control (no treatment) and pure PLGA control, indicating that PLGA alone has no antimicrobial activity. Moreover, thymol-loaded microparticles at a concentration of 5 mg/mL exhibited better antibacterial activity against *S. aureus* than *E. coli*. These findings corroborate those of a previous report that a higher antibacterial activity of nanofibers incorporating thymol was observed for *S. aureus* than *E. coli* [22]. The difference in antibacterial activity might be attributed to the different cell wall structure between Gram-positive and Gram-negative bacteria. Specifically, *E. coli* has a thin peptidoglycan layer and an outer layer of lipoproteins, lipopolysaccharides, and phospholipids, while the cell wall of *S. aureus* contains a peptidoglycan layer with many pores. Therefore, the porous structure could enhance the permeation of thymol into the bacterial cells [22].

**Figure 8:**
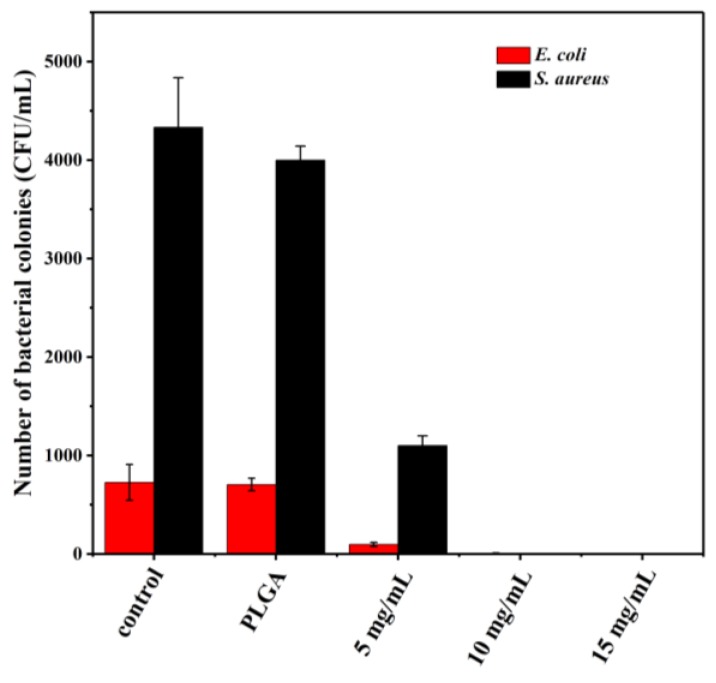
The number of bacterial colonies counted in milk treated with different amounts of thymol-loaded PLGA microparticles (0, 5, 10, and 15 mg/mL). Error bars indicate the standard deviation of the mean.

Milk and milk products are particularly susceptible to microbial contamination caused by *E. coli* and *S. aureus*. Thymol-loaded microparticles were added into naturally contaminated milk to test the applicability for antibacterial additives. The number of bacterial colonies counted in milk treated with different amounts of microparticles is presented in Figure 8. The initial amount of *S. aureus* colonies was more than that of *E. coli* in milk. The number of both bacteria in milk treated with thymol-loaded microparticles declined as the amount of microparticles increased, and their growth was totally suppressed when the thymol-loaded microparticles reached 10 mg/mL. The FDA Pasteurized Milk Ordinance (PMO) specifies limits for total bacterial counts and coliforms of 20,000 CFU/mL and 10 CFU/mL, respectively, throughout product shelf life in Grade A pasteurized fluid milk [27]. Our findings indicated that PLGA microparticles containing thymol could extend the shelf life of milk and meet the requirement for coliforms. The hydrophobic nature of thymol presents a challenge for microbial inhibition in aqueous media; however, it could be more effectively delivered to microorganisms after microencapsulation in PLGA [13]. This is because PLGA produces acid by-products during the degradation process, which increases its hydrophobicity and enables it to partition into the lipids of the microbial cell membrane or to bind to the hydrophobic regions of the proteins [28].

## 4. Conclusions

Thymol-loaded PLGA microparticles were prepared by a single emulsion solvent evaporation method. Microparticles containing thymol presented a spherical shape and smooth surface. Microencapsulation significantly improved thermal stability and inhibited the volatility of thymol. In vitro release profiles demonstrated an initial fast release followed by a slow and sustained release. Thymol-loaded microparticles showed strong antibacterial activity against *E. coli* and *S. aureus*. The effectiveness of its antibacterial activity was confirmed in a milk test, in which the growth of both bacteria was totally suppressed by microparticles containing thymol. Taken together, the results presented herein indicated that thymol-loaded microparticles show great potential for use as antimicrobial and preservation additives in food.

## References

[1] Miranda J.M., Mondragon A., Vazquez B.I., Fente C.A., Cepeda A., Franco C.M. (2009). Microbiological quality and antimicrobial resistance of *Escherichia coli* and *Staphylococcus aureus* isolated from conventional and organic “Arzua-Ulloa” cheese. CyTA-J. Food.

[2] Lopes N.A., Brandelli A. (2017). Nanostructures for delivery of natural antimicrobials in food. Crit. Rev. Food Sci..

[3] Marchese A., Orhan I.E., Daglia M., Barbieri R., Lorenzo A.D., Nabavi S.F., Gortzi O., Izadi M., Nabavi S.M. (2016). Antibacterial and antifungal activities of thymol: A brief review of the literature. Food Chem..

[4] Trombetta D., Castelli F., Sarpietro M.G., Venuti V., Cristani M., Daniele C., Saija A., Mazzanti G., Bisignano G. (2005). Mechanisms of antibacterial action of three monoterpenes. Antimicrob. Agents Chemother..

[5] Pan K., Chen H.Q., Davidson P.M., Zhong Q.X. (2014). Thymol nanoencapsulated by sodium caseinate: physical and antilisterial properties. J. Agr. Food Chem..

[6] Dima C., Cotârlet M., Alexe P., Dima S. (2014). Microencapsulation of essential oil of pimento [*Pimenta dioica* (L) Merr.] by chitosan/k-carrageenan complex coacervation method. Innov. Food Sci. Emerge..

[7] Benavides S., Cortés P., Parada J., Franco W. (2016). Development of alginate microspheres containing thyme essential oil using ionic gelation. Food Chem..

[8] Li N., Zhang Z.J., Li X.J., Li H.Z., Cui L.X., He D.L. (2018). Microcapsules biologically prepared using *Perilla frutescens* (L.) Britt. essential oil and their use for extension of fruit shelf life. J. Sci. Food Agr..

[9] Madene A., Jacquot M., Scher J., Desobry S. (2006). Flavour encapsulation and controlled release—A review. Int. J. Food Sci. Technol..

[10] Makadia H.K., Siegel S.J. (2011). Poly lactic-co-glycolic acid (PLGA) as biodegradable controlled drug delivery carrier. Polymers.

[11] Silva L.M., Hill L.E., Figueiredo E., Gomes C.L. (2014). Delivery of phytochemicals of tropical fruit by-products using poly (DL-lactide-co-glycolide) (PLGA) nanoparticles: synthesis, characterization, and antimicrobial activity. Food Chem..

[12] Aguilar-Tuesta S., Mamani-Navarro W., Espinoza-Silva C., Basilio-Atencio J., Condezo-Hoyos L. (2018). Microencapsulates of betacyanin from colored organic quinoa (*Chenopodium quinoa Willd*): Optimization, physicochemical characterization and accelerated storage stability. J. Sci. Food Agr..

[13] Hill L.E., Taylor T.M., Gomes C. (2013). Antimicrobial efficacy of poly (DL-lactide-co-glycolide) (PLGA) nanoparticles with entrapped cinnamon bark extract against *Listeria monocytogenes* and *Salmonella typhimurium*. J. Food Sci..

[14] Gomes C., Moreira R.G., Castell-Perez E. (2011). Poly (DL-lactide-co-glycolide) (PLGA) nanoparticles with entrapped trans-cinnamaldehyde and eugenol for antimicrobial delivery applications. J. Food Sci..

[15] Zhang Z.R., Wang X.Y., Li B.B., Hou Y.J., Cai Z.W., Yang J., Yi L. (2018). Paclitaxel-loaded PLGA microspheres with a novel morphology to facilitate drug delivery and antitumor efficiency. RSC Adv..

[16] (2016). Determination of Aerobic Plate Count in Foods.

[17] Han B.Z., Meng Y., Li M., Yang Y.X., Ren F.Z., Zeng Q.K., Nout M.J.R. (2007). A survey on the microbiological and chemical composition of buffalo milk in China. Food Control.

[18] Zhang Z.R., Wang X.Y., Li B.B., Hou Y.J., Yang J., Yi L. (2018). Development of a novel morphological paclitaxel-loaded PLGA microspheres for effective cancer therapy: in vitro and in vivo evaluations. Drug Deliv..

[19] Banerjee S., Chattopadhyay P., Ghosh A., Goyary D., Karmakar S., Veer V. (2013). Influence of process variables on essential oil microcapsule properties by carbohydrate polymer–protein blends. Carbohyd. Polym..

[20] Kumari S., Kumaraswamy R.V., Choudhary R.C., Sharma S.S., Pal A., Raliya R., Biswas P., Saharan V. (2018). Thymol nanoemulsion exhibits potential antibacterial activity against bacterial pustule disease and growth promotory effect on soybean. Sci. Rep..

[21] da Silva-Junior A.A., de Matos J.R., Formariz T.P., Rossanezi G., Scarpa M.V., do Egito E.S.T., de Oliveira A.G. (2009). Thermal behavior and stability of biodegradable spray-dried microparticles containing triamcinolone. Int. J. Pharm..

[22] Aytac Z., Ipek S., Durgun E., Tekinay T., Uyar T. (2017). Antibacterial electrospun zein nanofibrous web encapsulating thymol/cyclodextrin-inclusion complex for food packaging. Food Chem..

[23] Zolnik B.S., Leary P.E., Burgess D.J. (2006). Elevated temperature accelerated release testing of PLGA microspheres. J. Control. Release.

[24] Houchin M.L., Topp E.M. (2009). Physical properties of PLGA films during polymer degradation. J. Appl. Polym. Sci..

[25] Sansdrap P., Moes A.J. (1997). *In vitro* evaluation of the hydrolytic degradation of dispersed and aggregated poly (DL-lactide-co-glycolide) microspheres. J. Control. Release.

[26] Sikkema J., de Bont J.A.M., Poolman B. (1995). Mechanisms of membrane toxicity of hydrocarbons. Microbiol. Mol. Biol. R..

[27] (2011). “Standards for Grade “A” Milk and Milk Products,” in Grade “A” Pasteurized Milk Ordinance.

[28] Burt S. (2004). Essential oils: Their antibacterial properties and potential applications in foods—A review. Int. J. Food Microbio..

